# Supplementary material for: Blocking phospholamban with VHH intrabodies enhances contractility and relaxation in heart failure
Source: Nat Commun. 2022 May 31;13:3018. doi: 10.1038/s41467-022-29703-9 (PMC9156741; doi:10.1038/s41467-022-29703-9)
Supplement: Supplementary file 1 — Supplementary Information [file 41467_2022_29703_MOESM1_ESM.pdf]

Supplementary information

Blocking phospholamban with VHH intrabodies enhances contractility and relaxation in heart failure

Erwin De Genst[1,2*], Kylie S. Foo[2,3], Yao Xiao[2,3], Eduarde Rohner[2,3], Emma de Vries[1], Jesper Sohlmér[2,3], Nevin Witman[2], Alejandro Hidalgo[2,4], Terje R.S. Kolstad[5,6], William E. Louch[5,6], Susanne Pehrsson[4], Andrew Park[7], Yasuhiro Ikeda[7], Xidan Li[2], Lorenz M. Mayr[1,8], Kate Wickson[1], Karin Jennbacken[4], Kenny Hansson[4], Regina Fritsche-Danielson[4], James Hunt[1*] and Kenneth R. Chien[2,3*]

[1] Discovery Sciences, R&D, AstraZeneca, Cambridge, UK.

[2] Karolinska Institutet/AstraZeneca Integrated Cardio Metabolic Centre (KI/AZ ICMC), Department of Medicine, Karolinska Institutet, Huddinge, Sweden.

[3] Department of Cell and Molecular Biology, Karolinska Institutet, Stockholm, Sweden

[4] Bioscience Cardiovascular, Research and Early Development, Cardiovascular, Renal and Metabolism (CVRM), BioPharmaceuticals R&D, AstraZeneca, Gothenburg, Sweden.

[5] Institute for Experimental Medical Research, Oslo University Hospital and University of Oslo, Norway.

[6] K.G. Jebsen Centre for Cardiac Research, University of Oslo, Oslo, Norway.

[7] Biologics Engineering, R&D, AstraZeneca, One MedImmune Way, Gaithersburg, USA.

[8] Current address: Vector BioPharma AG, Aeschenvorstadt 36, 4051 Basel, Switzerland

*Corresponding Authors: kenneth.chien@ki.se, erwin.degenst@astrazeneca.com and james.hunt1@astrazeneca.com

Supplementary Figure 1

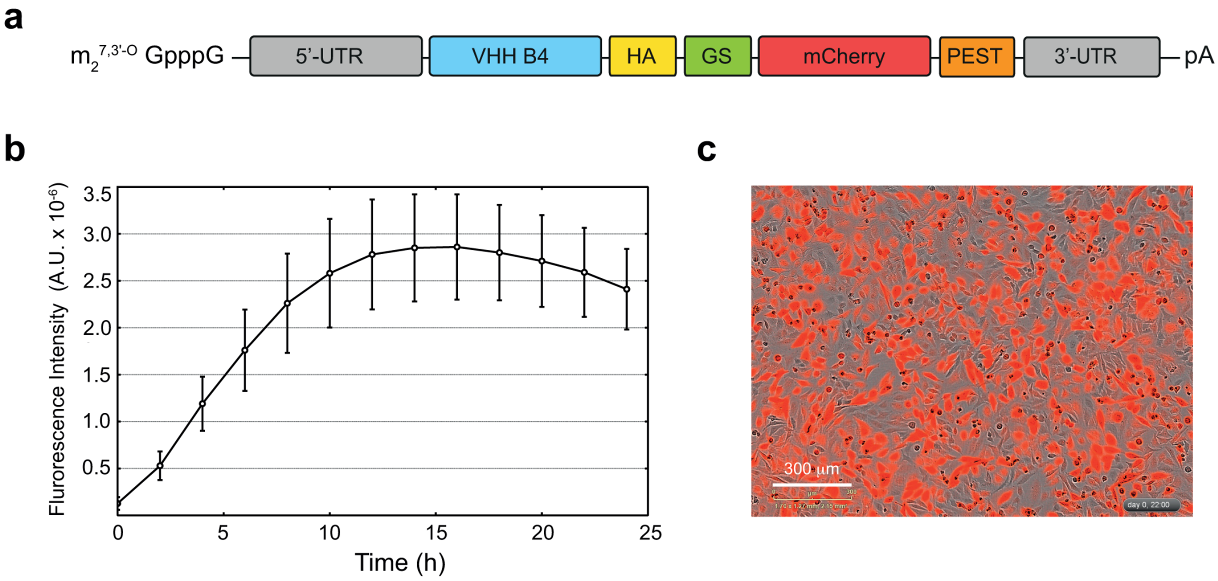

Figure 1: Time-lapse bright field and fluorescence imaging of HeLa cells transfected with VHH B4-HA-GS-mCherry-PEST using an incucyte imager. **a**) ModRNA construct, $m_2^{7,3'\text{-}O}$ GpppG is the ARCA CAP; 5'UTR is the 5' untranslated region, VHH is the sequence that codes for the intrabody, GS is a coding sequence for the linker sequence: GSAGSAAGSGEF, HA is the sequence encoding the hemagglutinin tag, mCherry represents the mCherry encoding sequence, PEST represents the sequence that codes for the PEST domain of mouse ornithine decarboxylase[1], 3' UTR is the 3' untranslated region and pA is the poly A tail. **b**) Average fluorescence of 3 wells of 96 well culture plate at different time points post-transfection. Error bars represent the standard error of the mean (SEM) of the fluorescence values for *n* = 3 replicate transfections. **c**) Fluorescence image of a representative well of cells transfected with VHH B4-HA-GS-mCherry modRNA at 22 hours post transfection. Two independent repetitions of the experiment provided similar results.

Supplementary Figure 2

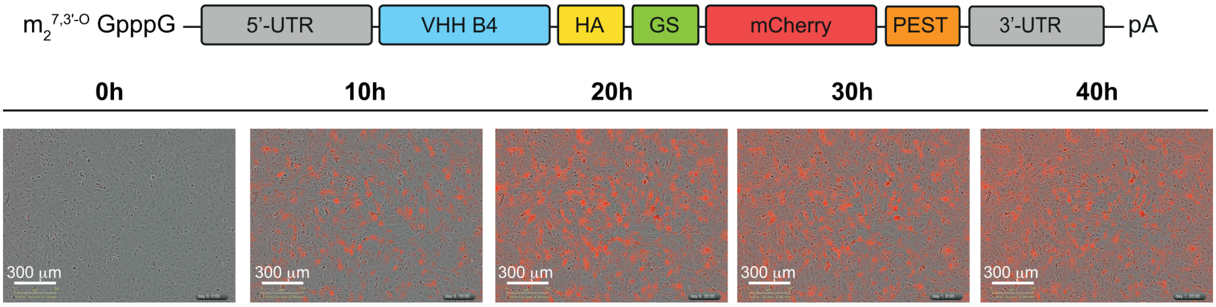

Figure 2: Time-lapse bright field and fluorescence imaging of induced pluripotent stem cell derived cardiomyocytes (iPS CM) cells transfected with VHH B4-HA-GS-mCherry and using an incucyte imager. The ModRNA construct, $m_2^{7,3'\text{-}O}$ GpppG is the ARCA CAP; 5'UTR is the 5' untranslated region, VHH is the sequence that codes for the intrabody, GS is a coding sequence for the linker sequence: GSAGSAAGSGEF, HA is the sequence encoding the hemagglutinin tag, mCherry represents the mCherry encoding sequence, PEST represents the sequence that codes for the PEST domain of mouse ornithine decarboxylase[1], 3' UTR is the 3' untranslated region and pA is the poly A tail. Fluorescence images of one of the representative wells of cells transfected with VHH B4-HA-GS-mCherry-PEST modRNA at 0, 10, 20, 30 and 40 hours post transfection are presented. This experiment has been independently replicated with the same construct and showed similar results and a similar experiment using constructs for which the mCherry-PEST sequence is replaced with a T2A mCherry sequence resulted in similar results.

Supplementary Figure 3

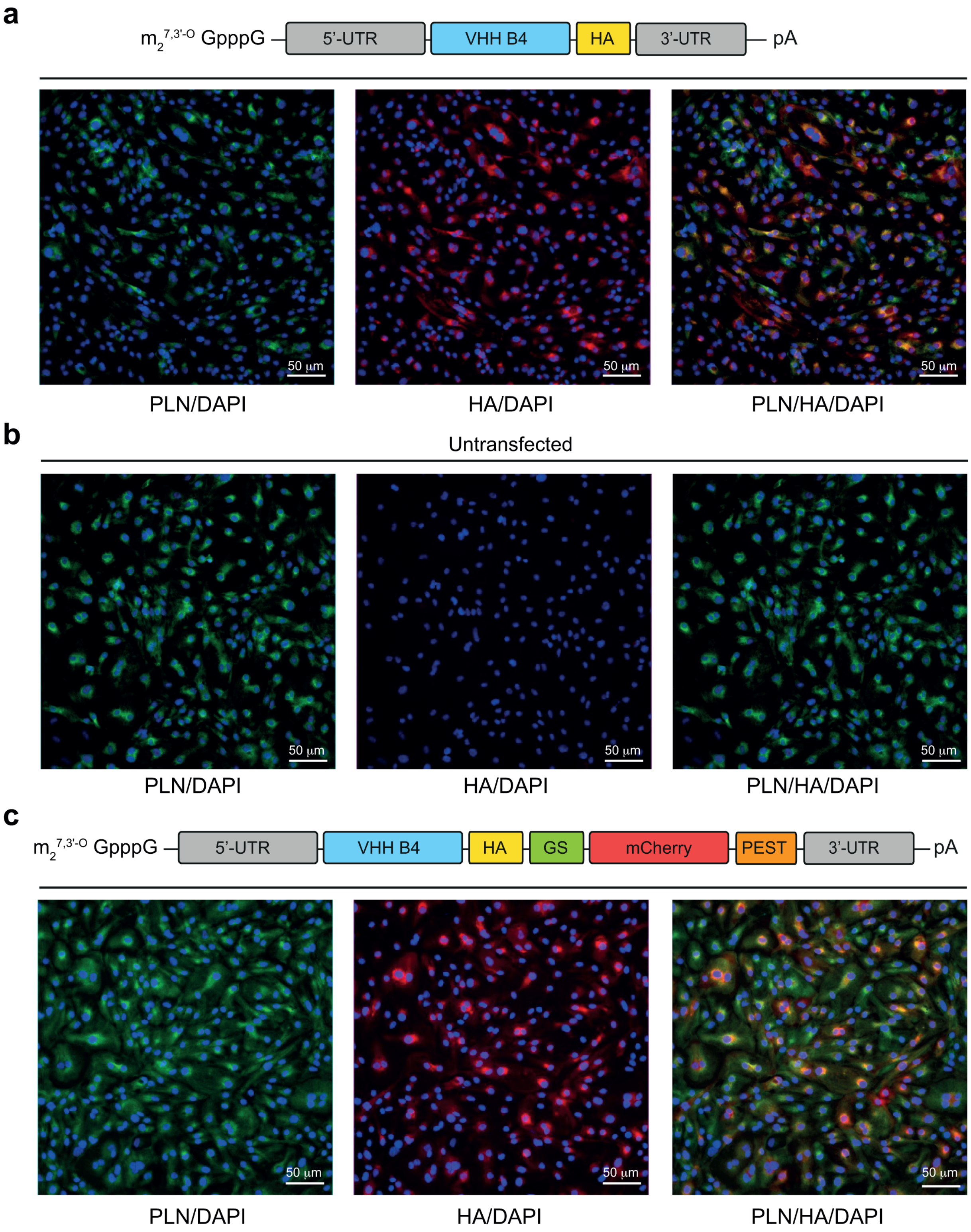

Figure 3: Immunofluorescence imaging of fixed and permeabilised iPS CM cells transfected for 24h with 400 ng modRNA encoding for VHH B4-HA or VHH B4-HA-GS-mCherry-PEST (modRNA constructs are indicated above the respective panels with elements identical to those described in supplementary figure 1 and 2). Cells were co-stained with anti-PLN (2D12), anti-HA (C29F4) and DAPI. Anti-Mouse secondary antibody Alexa Fluor 488

conjugate and anti-Rabbit Alexa Fluor 647 conjugate was used to detect the anti-PLN and anti-HA antibodies, respectively. **a)** Immunofluorescence images of fixed cells transfected with VHH B4-HA. **b)** Fluorescent images in overlay with the DAPI signal representing each channel: PLN (green), HA (red) and the PLN/HA overlay (green and red), are indicated at the bottom of their respective panel. **c)** Identical to **a)** except that cells were transfected with 400 ng of VHH B4-HA-GS-mCherry modRNA. Experiments depicted in a-c were not independently replicated.

Supplementary Figure 4

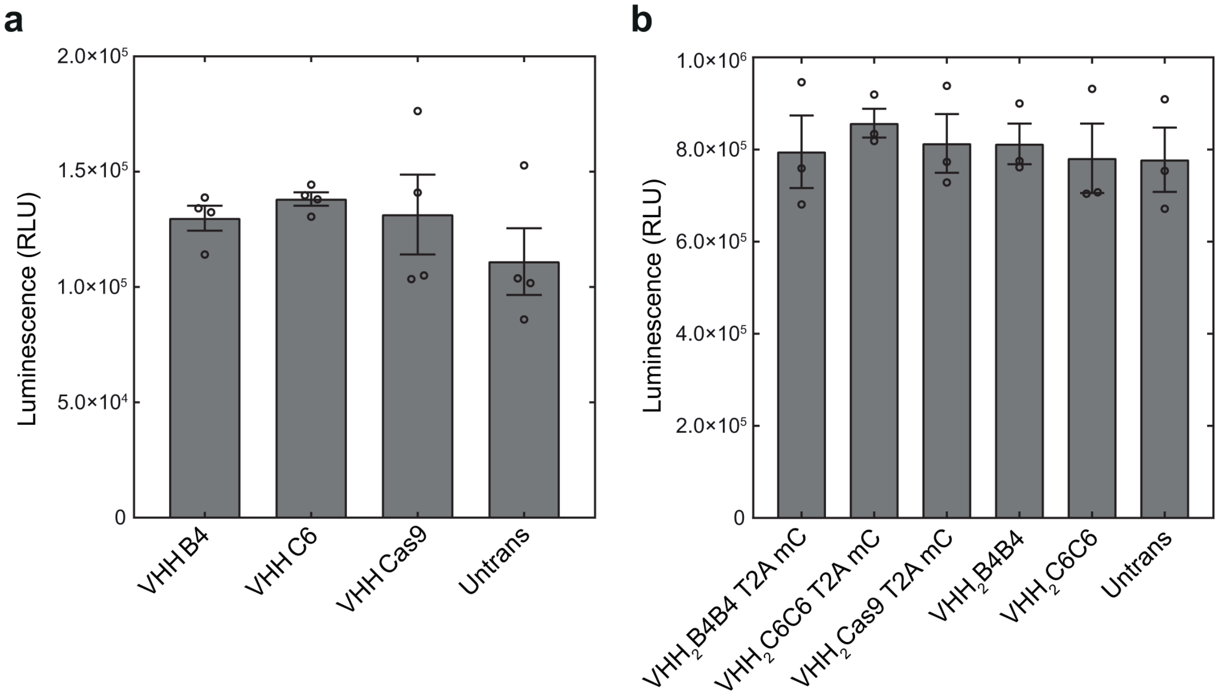

Figure 4. Cell viability of iPS cardiomyocytes after 36 hours of transfection. a) CellTiter-Glo® Luminescent Cell Viability Assay for human iPS derived cardiomyocytes transfected with VHH B4-HA, VHH C6-HA or VHH Cas9-HA (non specific control) compared to control cells. Mean values of 4 replicates are shown as bar graphs. Individual data points are plotted on the graph. Error bars indicate the standard error of the mean (SEM) between 3 transfections. Statistical significant differences with the control group were evaluated using one-way ANOVA using all data points and assuming normal distributions. Post-hoc two-sided *P*-values were calculated using Dunnett's method. No significant differences (*P*-value<0.05) between the treated groups versus control group were found. Average luminescence values and exact *P*-values can be found in Supplementary Table 6 **b)** CellTiter-Glo® 2.0 Luminescent Cell Viability Assay for human iPS derived cardiomyocytes after 36 hours of transfection with either VHH_2 B4B4-T2A-mCherry, VHH_2 C6C6-T2A-mCherry, VHH_2 Cas9-T2A-mCherry, VHH_2 B4B4-HA, VHH_2 C6C6-HA. Mean values of 3 replicate transfections are shown as bar graphs for and untransfected cells. Individual data points are plotted on the graph. Error bars indicate the standard error of the mean (SEM) between 3 transfections; statistical significant differences with the control group were evaluated using one-way ANOVA using all data points and assuming normal distributions. Post-hoc two-sided *P*-values were calculated using Dunnett's method. No significant differences (*P*-value<0.05) between the treated groups versus control group were found. Average luminescence values and exact *P*-values can be found in Supplementary Table 7. RLU relative luminescence units.

Supplementary Figure 5

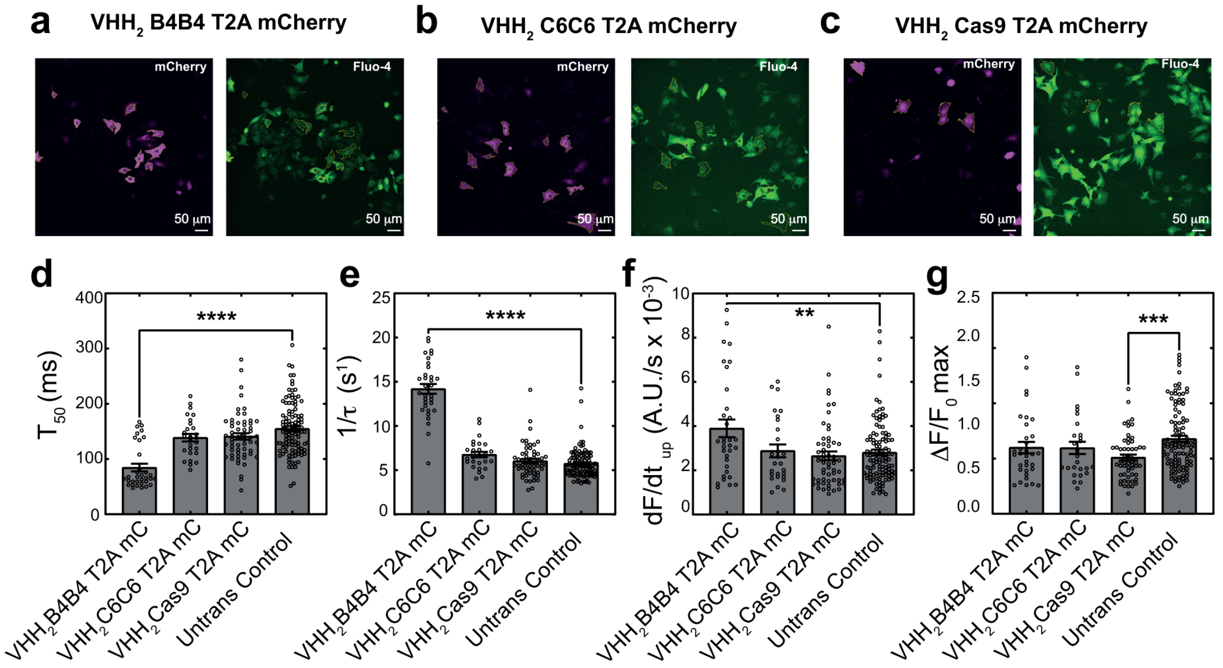

Figure 5. Transfection of rat neonatal cardiomyocytes with modRNA encoding for intrabody-T2A-mCherry genetic fusions and the effects on the Ca^{2+} dynamics of rat neonatal cardiomyocytes. **a-c)** Images of live transfected cells with the different modRNA encoded intrabodies, showing red mCherry fluorescence (left panel) and the corresponding Fluo-4 AM florescence imaging (right panels) (scale bars = 50 μm) **d)** T_{50} (peak half-width), **e)** $1/\tau$ (rate of transient decay), **f)** dF/dt_{up} (the upstroke velocity) and **g)** $\Delta F/F_{0max}$ (the amplitude of the Ca^{2+} transient) for the control and treated cells with modRNA encoding for the different intrabodies (indicated on the X-axis). VHH_2 B4B4-T2A-mCherry, n = 33, VHH_2 C6C6-T2A-mCherry, n = 27, VHH_2 Cas9-T2A-mCherry, n = 57, Untransfected control cells, n = 109. Bars represent the averages and error bars represent the standard error of the mean (SEM) of the sample set. Statistical significant differences with the control group are indicated and were evaluated using one-way ANOVA assuming normal distributions. Post-hoc two-sided P-values were calculated using Dunnett's method, and significance values for differences with the control group are indicated: **P-value<0.01, ***P-value<0.001, ****P-value<0.0001. Exact P-values can be found in Supplementary Table 8. A.U. arbitrary units. Source data are provided as a Source Data file.

Supplementary Figure 6

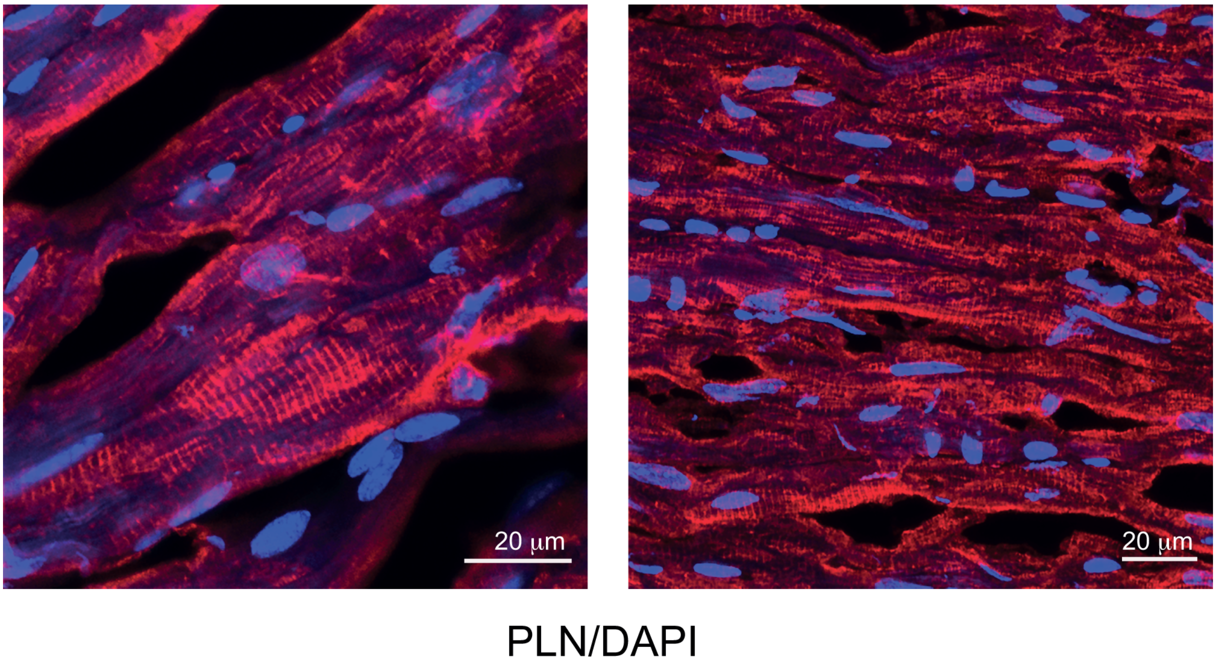

Figure 6. Staining of heart tissue with the PLN antibody 2D12 for validation as reagent for IF. The figure shows two different magnifications of mouse heart tissue stained with the 2D12 antibody and shows a very distinct striated pattern characteristic for the sarcoplasmic reticulum, in which the target protein PLN resides. Immunofluorescence staining were performed in n=3 mice, with 6-8 tissue sections per slide, 5-8 slides per mouse, experiments were repeated twice.

Supplementary Table 1: Kinetic and dissociation constants of the VHH PLN interaction

Supplementary Table 1						
	Peptide*	Temp (°C)	Fit	k_a ($M^{-1}s^{-1}$) * 10^{-5}	k_d (s^{-1})	K_d (nM)
VHH B4	PLN	25	1:1 Binding	5.73	0.025	43.7
	pS16 PLN	25	1:1 Binding (Steady state binding¶)	2.90	0.18	618 (682¶)
VHH_2 B4B4	PLN	25	1:1 Binding	7.17	1.1 10^{-4}	0.15
	pS16 PLN	25	1:1 Binding	51.7	5.2 10^{-3}	1.02
VHH C6	PLN	25	1:1 Binding	Nil	Nil	Nil
	pS16 PLN	25	1:1 Binding (Steady state binding)	4.96	0.33	662 (680¶)

*PLN, non-phosphorylated phospholamban peptide; pS16PLN, phospho-serine 16 phospholamban peptide. Peptides contain a biotin-HDPD moiety at the N-terminus for immobilisation on streptavidin-coated surfaces.

¶Values for K_d between brackets represent the value obtained by fitting the experimental equilibrium binding levels to a steady state binding model using the BIAevaluation software.

Supplementary Table 2: Intracellular expression and target engagement assays for intrabody candidates

Supplementary Table 2			
Intrabody	Specificity	Intracellular expression*	Target engagement¶
VHH B4	Pan specific	HeLa, iPS CM, RNHCM, hESC, murine mature CM	CoIP, IF
VHH_2 B4B4	Pan specific	hESC, RNHCM	CoIP, IF
VHH C6	pS16 specific	HeLa, iPS CM, RNHCM	IF
VHH_2 C6C6	pS16 specific	hESC, mature CM	IF

*IPS CM, induced pluripotent stem cell derived cardiomyocytes (Fuji Film Cellular Dynamics); RNHCM, day 6 old isolated rat neonatal cardiomyocytes; hESC, human embryonic stem cells; murine mature CM, Langendorff isolated mouse adult cardiomyocytes or adult murine cardiac tissue.
¶Co-IP, co-immunoprecipitation studies. For co-immunoprecipitation studies from cells not endogenously expression PLN, modRNA constructs encoding for PLN were co-transfected with intrabody modRNA. IF, immunofluorescence imaging.

Supplementary Table 3: Effects of intrabodies VHH B4-HA, VHH C6-HA and VHH_2 B4B4-HA on the Ca^{2+} transient of transfected neonatal cardiomyocytes

Supplementary Table 3						
	N	T_{50} (ms)	T_{75} (ms)*	dF/dt_{up} (A.U./s x 10^3)	$\Delta F/F_{0\ max}$	$1/\tau$ (s^{-1})
VHH B4	53	111 ± 13.4[¶]	210 ± 15.0[¶]	4.8 ± 0.42[¶]	0.88 ± 0.056[¶]	6.98 ± 0.39
VHH_2 B4B4	70	114 ± 12.2[¶]	214 ± 17.0[¶]	5.5 ± 0.46[¶]	1.01 ± 0.059[¶]	7.26 ± 0.52
VHH C6	129	188 ± 7.55[¶]	301 ± 10.9[¶]	3.1 ± 0.24[¶]	0.75 ± 0.060[¶]	5.12 ± 0.21
Untransfected	66	213 ± 8.73[¶]	330 ± 12.8[¶]	3.4 ± 0.21[¶]	1.02 ± 0.054[¶]	4.98 ± 0.15
P-values[a]						
Comparison		T_{50} (ms)	T_{75} (ms)	dF/dt_{up} (A.U./s x 10^3)	$\Delta F/F_{0\ max}$	$1/\tau$ (s^{-1})
Untransfected	VHH B4	<0.0001	<0.0001	<0.0001	0.0531	<0.0001
Untransfected	VHH_2 B4B4	<0.0001	<0.0001	<0.0001	0.9993	<0.0001
Untransfected	VHH C6	0.0012	0.0114	0.4971	<0.0001	0.9216
VHH C6	VHH B4	<0.0001	<0.0001	<0.0001	0.0303	<0.0001
VHH C6	VHH_2 B4B4	<0.0001	<0.0001	<0.0001	<0.0001	<0.0001
VHH B4	VHH_2 B4B4	0.9874	0.9889	0.0402	0.0654	0.7023

[¶] 95% confidence interval

[a]*P* values from two-sided Tukey-Kramer post- one-way ANOVA analysis for differences between groups

*Individual values of the measurements of T_{75} for the different groups are provided in Source Data File

Supplementary Table 4: Effects of intrabodies VHH_2 B4B4-HA, VHH_2 C6C6-HA and VHH_2 Cas9-HA on the Ca^{2+} transient of isolated adult cardiomyocytes from WT C57BL/6

Supplementary Table 4								
	N		$1/\tau$ (s^{-1})		T_{50} (ms)		$\Delta F/F_{0\ max}$	
	1 Hz	4 Hz	1 Hz	4 Hz	1Hz	4Hz	1Hz	4Hz
VHH_2 B4B4	14	6	22.7 ± 2.70[¶]	26.1 ± 3.40[¶]	85.0 ± 6.10[¶]	72 ± 6.4[¶]	2.4 ± 0.52[¶]	1.7± 0.45[¶]
VHH_2 C6C6	13	5	186 ± 7.80[¶]	186 ± 7.80[¶]	175 ± 27.8[¶]	298 ± 12.0[¶]	1.7 ± 0.46[¶]	1.02 ± 0.35[¶]
VHH_2 Cas9	6	4	10.9 ± 3.60[¶]	13.5 ± 5.00[¶]	130 ± 25.6[¶]	94 ± 7.6[¶]	2.4 ± 0.80[¶]	1.3 ± 0.46[¶]
Untransfected	52	38	9.50 ± 1.00[¶]	13.0 ± 1.10[¶]	145 ± 7.90[¶]	97 ± 3.1[¶]	2.6 ± 0.22[¶]	1.7 ± 0.19[¶]
P-values[a]								
			$1/\tau$ (s^{-1})		T_{50} (ms)		$\Delta F/F_{0\ max}$	
Comparison			1 Hz	4 Hz	1Hz	4Hz	1Hz	4Hz
VHH_2 B4B4	VHH_2 C6C6		<0.0001	<0.0001	<0.0001	<0.0001	0.1472	0.2058
VHH_2 B4B4	VHH_2 Cas9		<0.0001	<0.0001	0.0234	0.0070	>0.9999	0.6141
VHH_2 B4B4	Untransfected		<0.0001	<0.0001	<0.0001	<0.0001	0.8507	0.9932
VHH_2 C6C6	VHH_2 Cas9		0.2782	0.5721	0.0237	0.3111	0.3265	0.9261
VHH_2 C6C6	Untransfected		0.3204	0.4270	0.0140	0.2828	0.0058	0.1155
VHH_2 Cas9	Untransfected		0.8398	0.9935	0.6886	0.9332	0.9482	0.5794

[¶] 95% confidence interval

[a]*P* values from two-sided Tukey-Kramer post- one-way ANOVA analysis for differences between groups

Supplementary Table 5: Hemodynamic parameters of $MLP^{-/-}$ mice treated with AAV9 encoding for VHH_2 B4B4 T2A ZsGreen or AAV9 ZsGreen control

Supplementary Table 5					
	N	d*P*/dT_{max} (mmHg/s)	d*P*/dT_{min} (mmHg/s)	τ (ms)
VHH_2 B4B4	5	5960 ± 767.0¶	- 4990 ± 615.0¶	6.1 ± 1.2¶
ZsGreen	7	4960 ± 380.0¶	- 3760 ± 322.0¶	8.9 ± 1.2¶
**P*-values					
Comparison		d*P*/dT_{max} (mmHg/s)	d*P*/dT_{min} (mmHg/s)	τ (ms)
ZsGreen	VHH_2 B4B4	0.037	0.0038	0.011

¶ 95% confidence interval

**P* values from two-tailed unpaired *t*-Test between VHH_2 B4B4 ZsGreen AAV9 treated mice and the control group (AAV9 ZsGreen)

Supplementary Table 6: Luminescent Cell Viability Assay for human iPS derived cardiomyocytes transfected with VHH B4-HA, VHH C6-HA or VHH Cas9-HA

Supplementary Table 6		
	N	Average Luminosity (RLU)
VHH B4	4	129846 ± 10600[¶]
VHH C6	4	138138 ± 5660.0[¶]
VHH Cas9	4	131403 ± 33900[¶]
Untransfected	4	111024 ± 28300[¶]
P-values[a]		
Comparison		Average Luminosity (RLU)
Untransfected	VHH B4	0.5481
Untransfected	VHH C6	0.2815
Untransfected	VHH Cas9	0.4900

[¶] 95% confidence interval
[a]*P* values from two-sided Dunett post- one-way ANOVA analysis for differences with Untransfected cells

Supplementary Table 7: Luminescent Cell Viability Assay for human iPS derived cardiomyocytes transfected with VHH_2 B4B4-T2A-mCherry, VHH_2 C6C6-T2A-mCherry, VHH_2 Cas9-T2A-mCherry, VHH_2 B4B4-HA, VHH_2 C6C6-HA

Supplementary Table 7		
	N	Average Luminosity (RLU)
VHH_2 B4B4 T2A mC	3	795920 ± 154000[¶]
VHH_2 C6C6 T2A mC	3	857840 ± 61300[¶]
VHH_2 Cas9 T2A mC	3	813827 ± 125000[¶]
VHH_2 B4B4	3	812787 ± 86400[¶]
VHH_2 C6C6	3	781613 ± 148000[¶]
Untransfected	3	778600 ± 137000[¶]
P-values[a]		
Comparison		Average Luminosity (RLU)
Untransfected	VHH_2 B4B4 T2A mC	0.9997
Untransfected	VHH_2 C6C6 T2A mC	0.8382
Untransfected	VHH_2 Cas9 T2A mC	0.993
Untransfected	VHH_2 B4B4	0.9939
Untransfected	VHH_2 C6C6	>0.9999

[¶] 95% confidence interval

[a] *P* values from two-sided Dunett post- one-way ANOVA analysis for differences with Untransfected cells

Supplementary Table 8: Effects of VHH_2 B4B4-T2A-mCherry, VHH_2 C6C6-T2A-mCherry, VHH_2 Cas9-T2A-mCherry intrabodies on the Ca^{2+} transient of transfected neonatal cardiomyocytes.

Supplementary Table 8						
	N	T_{50} (ms)	T_{75} (ms)*	dF/dt_{up} (A.U./s x 10^3)	$\Delta F/F_{0\ max}$	$1/\tau$ (s^{-1})
VHH_2 B4B4 T2A-mC	33	84.5 ± 13.5[¶]	136.9 ± 20.2[¶]	3.90 ± 0.79[¶]	0.60 ± 0.10[¶]	14.2 ± 1.07
VHH_2 C6C6 T2A-mC	27	138.6 ±13.4[¶]	235.6 ± 22.5[¶]	2.88 ± 0.57[¶]	0.60 ± 0.11[¶]	6.75 ± 0.62
VHH_2 Cas9-T2A mC	57	141.0 ± 11.3 [¶]	251.3 ± 19.9[¶]	2.65 ± 0.39[¶]	0.51 ± 0.05[¶]	6.02 ± 0.49
Untransfected	109	154.8 ± 8.87[¶]	265.7 ± 13.7[¶]	2.80 ± 0.26[¶]	0.68 ± 0.05[¶]	5.73 ± 0.30
P-values (Dunnett)[a]						
Comparison		T_{50} (ms)	T_{75} (ms)	dF/dt_{up} (A.U./s x 10^3)	$\Delta F/F_{0\ max}$	$1/\tau$ (s^{-1})
Untransfected	VHH_2 B4B4	<0.0001	<0.0001	0.0018	0.3506	<0.0001
Untransfected	VHH_2 C6C6	0.2332	0.1335	0.9933	0.3893	0.0470
Untransfected	VHH_2 Cas9	0.1539	0.4983	0.9102	0.0005	0.7287

[¶] 95% confidence interval

[a]*P* values from two-sided Dunett post- one-way ANOVA analysis for differences with Untransfected cells

* Individual values of the measurements of T_{75} for the different groups are provided in Source Data File

Supplementary References

1. Ghoda, L., Wetters, T. van D., Macrae, M., Ascherman, D. & Coffino, P. Prevention of rapid intracellular degradation of ODC by a carboxyl-terminal truncation. *Science* **243**, 1493–1495 (1989).
