# Supplementary material for: Blocking phospholamban with VHH intrabodies enhances contractility and relaxation in heart failure
Source: Nat Commun. 2022 May 31;13:3018. doi: 10.1038/s41467-022-29703-9 (PMC9156741; doi:10.1038/s41467-022-29703-9)
Supplement: Supplementary file 3 — Reporting Summary [file 41467_2022_29703_MOESM3_ESM.pdf]

nature portfolio

Corresponding author(s): Erwin De Genst, James Hunt, Kenneth R Chien

Last updated by author(s): Feb 21, 2022

Reporting Summary

Nature Portfolio wishes to improve the reproducibility of the work that we publish. This form provides structure for consistency and transparency in reporting. For further information on Nature Portfolio policies, see our Editorial Policies and the Editorial Policy Checklist.

Statistics

For all statistical analyses, confirm that the following items are present in the figure legend, table legend, main text, or Methods section.

n/a	Confirmed	
☐	☒	The exact sample size (*n*) for each experimental group/condition, given as a discrete number and unit of measurement
☐	☒	A statement on whether measurements were taken from distinct samples or whether the same sample was measured repeatedly
☐	☒	The statistical test(s) used AND whether they are one- or two-sided<br>*Only common tests should be described solely by name; describe more complex techniques in the Methods section.*
☒	☐	A description of all covariates tested
☐	☒	A description of any assumptions or corrections, such as tests of normality and adjustment for multiple comparisons
☐	☒	A full description of the statistical parameters including central tendency (e.g. means) or other basic estimates (e.g. regression coefficient) AND variation (e.g. standard deviation) or associated estimates of uncertainty (e.g. confidence intervals)
☐	☒	For null hypothesis testing, the test statistic (e.g. *F*, *t*, *r*) with confidence intervals, effect sizes, degrees of freedom and *P* value noted<br>*Give P values as exact values whenever suitable.*
☒	☐	For Bayesian analysis, information on the choice of priors and Markov chain Monte Carlo settings
☒	☐	For hierarchical and complex designs, identification of the appropriate level for tests and full reporting of outcomes
☒	☐	Estimates of effect sizes (e.g. Cohen's *d*, Pearson's *r*), indicating how they were calculated

Our web collection on statistics for biologists contains articles on many of the points above.

Software and code

Policy information about availability of computer code

Data collection	NIS elements 5.11.01 was used for Fluo-4 Calcium imaging, Incucyte Zoom 2016A for time lapse imaging of cell-line transfection, Biacore control software for T200 2.0 for affinity measurements, Hemodynamic parameters, dP/dt max, dP/dt min, LVPed, LVPes, heart rate and Tau were measured via a 1-Fr pressure catheter (PVR-1035, Millar Instruments, Houston, Texas, US) and hemodynamics was measured via the MPVS-Ultra Single Segment Foundation System (Millar, ADInstruments, US), Vevo LAB software version 3.2.6 (FUJIFILM VisualSonics) was used for echocardiography. LabChart v8.1.16 was used to record the respiration rate of the mice as a pressure readout.
Data analysis	Biacore evaluation software for T200 v 1.0 was used for fitting kinetic and binding data for the antibody fragments binding to immobilised PLN peptides. NIS elements 5.11.01 AR and Fiji Image J 2.0.0 were used for calcium imaging analysis. Statistical analyses were carried out using graph-pad prism 9.0.0. WinEDR (v.3.8.9) was used for measurement of the tau values of the calcium transients of rat neonatal cardiomyocytes.

For manuscripts utilizing custom algorithms or software that are central to the research but not yet described in published literature, software must be made available to editors and reviewers. We strongly encourage code deposition in a community repository (e.g. GitHub). See the Nature Portfolio guidelines for submitting code & software for further information.

Data

Policy information about availability of data

All manuscripts must include a data availability statement. This statement should provide the following information, where applicable:

- Accession codes, unique identifiers, or web links for publicly available datasets
- A description of any restrictions on data availability
- For clinical datasets or third party data, please ensure that the statement adheres to our policy

Data necessary to interpret, verify and extend the research are available in the main article file, the supplementary materials and the Source data file. All other raw data can be obtained from the authors upon reasonable request. Source data are provided with this paper.

Field-specific reporting

Please select the one below that is the best fit for your research. If you are not sure, read the appropriate sections before making your selection.

☒ Life sciences ☐ Behavioural & social sciences ☐ Ecological, evolutionary & environmental sciences

For a reference copy of the document with all sections, see nature.com/documents/nr-reporting-summary-flat.pdf

Life sciences study design

All studies must disclose on these points even when the disclosure is negative.

Sample size	1) Intrabody expression, co-IP experiments, and Ca2+ flux experiments in rat neonatal cardiomyocytes: For each experiment a litter of male day 6 rat pups (6-12 pups) were used to isolate rat neonatal cardiomyocytes, which were then pooled and cultured. Ca2+ flux measurements in Fig 3 were performed in imaging culture dishes and using 5-6 fields of view chosen at random locations in the well. No statistical method was performed as effect size was unknown. Post calcium imaging immunofluorescence staining was performed for all samples at 3 different locations in the dish.<br><br>2) In vivo expression of intrabodies in myocardium. Our aim was not to provide any quantitative assessment of the expression levels, but merely qualitative assessment and demonstration of the expression kinetics of the modRNA injected in the live murine heart. Therefore we chose the number of animals per group as a compromise to minimise animal use and to capture a qualitative picture of the biological variability. A total of 12 mice were used per group, whereby 6 would be sacrificed after 24 h and the 6 following were sacrificed after 72h. Half of the mice were used for western blot analysis and the other half were used for tissue sectioning and immunofluorescence imaging.<br><br>3) For the Ca2+ flux experiments, data from as many as possible transfected live cells were collected from Langendorff perfusion experiments over a number of animals (VHH2 B4B4 T2A mcherry n = 6, VHH C6C6 T2a mCherry n = 3, VHH Cas9 mCherry n = 2). Data from different preps/ animals were pooled per group. In addition, data from non-transfected cells within each prep were collected and pooled and assigned to the control group. We used this approach due to the technical challenges associated to the isolation of live transfected cells from the myocardium after ModRNA using Langendorff perfusion, only a small number of transfected cells were collected per animal.<br><br>4) For the hemodynamic experiments in MLP KO mice the sample size was determined using a power calculation based on the ability to detect a 8 % effect increase (based on different studies) with 80% confidence at an alpha value of 0.5, leading to a sample size of 5 mice. In the the end 7 mice were available per group. Tissue samples (heart, liver and quadriceps) from all mice, were collected and analysed on Western blot.<br><br>5) For the transfection experiments described in Supplementary Fig 1 and 2, three wells of a 96 well plate of HeLa cells (Supplementary Fig 1) or iPS derived cardiomyocytes (Supplementary Fig 2) was used to monitor variability between plated wells. No statistics were calculated as only a qualitative assessment of expression kinetics was required.<br><br>6) For the cell toxicity measurements depicted in Supplementary Fig 4 a, no sample size determination was performed and we limited ourselves to a qualitative assessment of cell toxicity after transfection of our modRNA constructs in a cardiac cell model. We added 3-4 technical replicates to allow an estimate of well to well variability.<br><br>7) Sample sizes in Supplementary Fig. 5 were determined by the collection of as many as possible viable transfected cells. A rough estimate of sample sizes needed to detect changes similar to those observed in fig 3 was calculated post-hoc and concluded to have sufficient power (>80% confidence at alpha 0.05) for the detection of similar effect sizes and standard deviations
Data exclusions	1) Ca2+ flux measurements in rat neonatal cardiomyocytes: cells displaying more or less than 1 calcium transient per second were excluded from the the analysis. Suspected outliers were manually checked and only removed when identified as errors in entry or measurement errors or unreliable measurements and mentioned in the figure legend. All remaining data points were included in the statistical analyses.<br>2) Ca2+ flux measurements in mouse adult cardiomyocytes: Cells with ΔF/F0max below 100 RU and those with aberrant behavior (aberrant pacing or the presence of extensive calcium waves or extensive spontaneous twitching) were rejected from the analysis. All remaining data points were included in the statistical analyses.<br>3) In the MLP KO hemodynamic study, two mice from the 7 individual mice in the VHH2B4B4 group were not able to be measured due to resource constraints. All remaining 5 data points were included in the statistical analyses.
Replication	Due to ethical reasons (to minimise animal experiments), replication was not performed for the hemodynamic measurements as our power

calculation resulting in the study sample size provided enough confidence.
intracardial expression profiling was carried out on 12 mice per modRNA (4 groups of 3) in total on different days and using different modRNA preps. Therefore we considered each mouse to represent an independent experiment. Therefore expression profiling by westernblotting was carried out 3 times per time point and Immunofluorescence experiement were carried out 2 times, only for the 24h timepoint, which is the time around maximal expression of the transfected modRNA.
The rat neonatal cardiomyocyte calcium flux experiment in fig 3 was not repeated. However, two independent calcium flux measurements with different intrabody constructs (including a T2A mCherry tag) were performed and were strongly supportive of the results of the larger set of untagged intrabody constructs (Fig 3, Supplementary Fig. 5).
The immunofluorescence measurements in figure 3i was not repeated. Staining of other cultured cardiomyocytes (hiPS CM, Supplementary Fig. 3) showed similar staining patterns and therfore are supportive of each other
All westernblotting was repeated at least 3 times and showed identical results.
Biacore measurements were performed twice and showed identical results
hiPS CM Cell viability studies after modRNA transfection has been measured only once for every construct. Due to the high cost of these hiPS CM we have limited to a qualitative assessment of intrabody toxicity. Although in the two different experiments different constructs were used, the overall picture of the absence of cyto toxicity due to intrabody expression can be confidently stated to be minimal if any.
Immunofluorescence staining shown in Supplementary Figure 6 was performed in n = 3 mice, with 6-8 tissue sections per slide, 5-8 slides per mouse, experiments were repeated twice.

Randomization

In all animal studies, including intracardial modRNA injections, allocation animals was randomised by weight and age. For the hemodynamic study off AAV9 transduced MLP KO mice, randomisation also included the measured ejection fraction before transduction.
The sequence order of the modRNA constructs to be injected intracardially was chosen randomly
Rat neonatal cardiomyocytes were isolated typically from 10-12 rat pups and pooled before plating and culture.

Blinding

For the hemodynamic measurements of AAV9 transduced MLP KO mice, the investigators, handling the mice, administering the treatment or vehicle and performing measurement and analysis were blinded.
For the in vitro experiments, blinding was not feasible due to resource constraints. However, microscopic analysis for IHC was performed in batches, and the investigators were not directly aware of the treatment conditions.

Reporting for specific materials, systems and methods

We require information from authors about some types of materials, experimental systems and methods used in many studies. Here, indicate whether each material, system or method listed is relevant to your study. If you are not sure if a list item applies to your research, read the appropriate section before selecting a response.

Materials & experimental systems

n/a	Involved in the study
☐	☒ Antibodies
☐	☒ Eukaryotic cell lines
☒	☐ Palaeontology and archaeology
☐	☒ Animals and other organisms
☒	☐ Human research participants
☒	☐ Clinical data
☒	☐ Dual use research of concern

Methods

n/a	Involved in the study
☒	☐ ChIP-seq
☒	☐ Flow cytometry
☒	☐ MRI-based neuroimaging

Antibodies

Antibodies used

mouse Anti-β-tubulin antibody (Thermo Scientific, USA, cat no MA5-16308-HRP)
mouse anti PLN 2D12 (Thermo Fisher Scientific, USA, cat no MA3-922)
rat anti-HA Fab 3F10 (Sigma, Germany, cat. no 12158167001)
rabbit anti-HA antibody C29F4 (Cell Signalling Technology, USA, cat no 3724)
mouse anti SERCA2a (MA3-910, Thermo Fisher Scientific, USA, cat no MA3-910)
Cofilin (D3F9) XP® Rabbit mAb (HRP Conjugate, cat no 8503)
anti-VHH antibody (MonoRabTM Rabbit anti-camelid VHH cocktail, Genscript, USA, cat no Cat. No. A02016)
anti GAPDH (14C10) Rabbit mAb (Cell Signalling Technology, USA, cat no #2118)
anti-rabbit-HRP (Cell Signalling Technology, USA, cat no #7074)
anti-mouse-HRP (Cell Signalling Technology, USA, cat no #7076)

Validation

All used antibodies concern commercially available and extensively tested antibodies. We refer to the website of the manufacturer for validation experiments and extensive lists of publications.

ThermoFisher MA5-16308-HRP, https://www.thermofisher.com/antibody/product/beta-Tubulin-Loading-Control-Antibody-clone-BT7R-Monoclonal/MA5-16308-HRP

ThermoFisher MA3-922: https://www.thermofisher.com/antibody/product/Phospholamban-Antibody-clone-2D12-Monoclonal/MA3-922

ThermoFisher MA3-910: https://www.thermofisher.com/antibody/product/SERCA2-ATPase-Antibody-clone-2A7-A1-Monoclonal/

MA3-919

Invitrogen MAS-15738: https://www.thermofisher.com/antibody/product/GAPDH-Loading-Control-Antibody-clone-GA1RMonoclonal/

Sigma-Aldrich, Anti-HA-Biotin, High Affinity (3F10) https://www.sigmaaldrich.com/GB/en/product/roche/12158167001

Cell signalling Technology C29F4 https://www.cellsignal.co.uk/products/primary-antibodies/ha-tag-c29f4-rabbit-mab/3724

ThermoFisher MA3-910: https://www.thermofisher.com/antibody/product/SERCA2-ATPase-Antibody-clone-IID8-Monoclonal/MA3-910

Cell Signalling Technology #8503 https://www.cellsignal.de/products/antibody-conjugates/cofilin-d3f9-xp-rabbit-mab-hrp-conjugate/
Genscript anti-VHH antibody Cat. No. A02016 https://www.genscript.com/antibody/A02016-MonoRab_Rabbit_Anti_Camelid_VHH_Cocktail_HRP_.html

Cell Signalling Technology anti GAPDH (14C10) Rabbit mAb (Cell Signalling Technology, USA) #2118:https://www.cellsignal.co.uk/products/primary-antibodies/gapdh-14c10-rabbit-mab/2118

anti-rabbit-HRP (Cell Signalling Technology, USA, cat no #7074) & anti-mouse-HRP (Cell Signalling Technology, USA, cat no #7076):
https://www.cellsignal.co.uk/products/secondary-antibodies/anti-rabbit-igg-hrp-linked-antibody/7074
https://www.cellsignal.co.uk/products/secondary-antibodies/anti-mouse-igg-hrp-linked-antibody/7076

Eukaryotic cell lines

Policy information about cell lines

Cell line source(s)	Hela (ATCC, CCL-2), iCell Cardiomyocytes2 (FujiFilm Cellular Dynamics), HES-3 embryonic stem cells (WiCell), AD-293 cells were purchased from Agilent (https://www.agilent.com/en/product/protein-expression/protein-expression-vectors-kits/viral-mediated-delivery-systems/ad-293-cells-232994#support)
Authentication	Cell lines were obtained from commercial source and authenticated via STR testing
Mycoplasma contamination	All cell lines tested negative for mycoplasma contamination
Commonly misidentified lines (See ICLAC register)	No commonly misidentified cell lines were used in this study

Animals and other organisms

Policy information about studies involving animals; ARRIVE guidelines recommended for reporting animal research

Laboratory animals	1) Rat neonatal cardiomyocytes were isolated from 6 day old male Sprague-Dawley rat pups<br>2) Mice for intrabody expression and the preperation of mature cardiomyocytes were male C57BL/6 between 12-20 months of age<br>3) Male muscle lim protein-deficient mice (MLP(-/-)) (MLP KO), 14-17 weeks of age, on black Swiss background, were purchased from Taconic (Denmark)
Wild animals	no wild animals were used in this study
Field-collected samples	no field collected samples were used in this study
Ethics oversight	Animal work at Karolinska Institutet was carried out in accordance with the institutional guidelines, and all animal experiments were approved by the local ethics committee (Stockholm, Sweden) in accordance with the Animal Protection Law, the Animal Protection Regulation, and the Regulation of the Swedish National Board for laboratory animals. Animal work at AstraZeneca was performed in accordance with the National Institute of Health (NIH) guidelines for use of experimental animals and the study protocol was approved by the Animal Ethics Committee at Gothenburg University (Gothenburg Ethical Review Board number Ea001173-2017).

Note that full information on the approval of the study protocol must also be provided in the manuscript.
